# Reprogramming of the Ethanol Stress Response in Saccharomyces cerevisiae by the Transcription Factor Znf1 and Its Effect on the Biosynthesis of Glycerol and Ethanol

**DOI:** 10.1128/AEM.00588-21

**Published:** 2021-07-27

**Authors:** Wiwan Samakkarn, Khanok Ratanakhanokchai, Nitnipa Soontorngun

**Affiliations:** a King Mongkut’s University of Technology Thonburi, Division of Biochemical Technology, School of Bioresources and Technology, Bangkok, Thailand; b Pilot Plant Development and Training Institute, King Mongkut’s University of Technology Thonburi, Bangkok, Thailand; Nanjing Agricultural University

**Keywords:** ethanol stress response, fermentation, glycerol, *S. cerevisiae*, transcription factor, unfolded protein response, Znf1, yeasts

## Abstract

High ethanol levels can severely inhibit the growth of yeast cells and fermentation productivity. The ethanologenic yeast Saccharomyces cerevisiae activates several well-defined cellular mechanisms of ethanol stress response (ESR); however, the involved regulatory control remains to be characterized. Here, we report a new transcription factor of ethanol stress adaptation called Znf1. It plays a central role in ESR by activating genes for glycerol and fatty acid production (*GUP1*, *GPP1*, *GPP2*, *GPD1*, *GAT1*, and *OLE1*) to preserve plasma membrane integrity. Importantly, Znf1 also activates genes implicated in cell wall biosynthesis (*FKS1*, *SED1*, and *SMI1*) and in the unfolded protein response (*HSP30*, *HSP104*, *KAR1*, and *LHS1*) to protect cells from proteotoxic stress. The *znf1*Δ strain displays increased sensitivity to ethanol, the endoplasmic reticulum (ER) stressor β-mercaptoethanol, and the cell wall-perturbing agent calcofluor white. To compensate for a defective cell wall, the strain lacking *ZNF1* or its target *SMI1* displays increased glycerol levels of 19.6% and 27.7%, respectively. Znf1 collectively regulates an intricate network of target genes essential for growth, protein refolding, and production of key metabolites. Overexpression of *ZNF1* not only confers tolerance to high ethanol levels but also increases ethanol production by 4.6% (8.43 g/liter) or 2.8% (75.78 g/liter) when 2% or 20% (wt/vol) glucose, respectively, is used as a substrate, compared to that of the wild-type strain. The mutually stress-responsive transcription factors Msn2/4, Hsf1, and Yap1 are associated with some promoters of Znf1’s target genes to promote ethanol stress tolerance. In conclusion, this work implicates the novel regulator Znf1 in coordinating expression of ESR genes and illuminates the unifying transcriptional reprogramming during alcoholic fermentation.

**IMPORTANCE** The yeast S. cerevisiae is a major microbe that is widely used in food and nonfood industries. However, accumulation of ethanol has a negative effect on its growth and limits ethanol production. The Znf1 transcription factor has been implicated as a key regulator of glycolysis and gluconeogenesis in the utilization of different carbon sources, including glucose, the most abundant sugar on earth, and nonfermentable substrates. Here, the role of Znf1 in ethanol stress response is defined. Znf1 actively reprograms expression of genes linked to the unfolded protein response (UPR), heat shock response, glycerol and carbohydrate metabolism, and biosynthesis of cell membrane and cell wall components. A complex interplay among transcription factors of ESR indicates transcriptional fine-tuning as the main mechanism of stress adaptation, and Znf1 plays a major regulatory role in the coordination. Understanding the adaptive ethanol stress mechanism is crucial to engineering robust yeast strains for enhanced stress tolerance or increased ethanol production.

## INTRODUCTION

The stress tolerance ability of yeast strains is crucial for the development of cell factories for industrial fermentation of biofuel and biochemicals. Osmotic or sugar stress, heat shock, and ethanol stress within the fermentation environment result in growth inhibition and increased death of yeast cells (1, 2), adversely affecting fermentation performance (3, 80). To improve tolerance to the harsh fermentation environment and maintain economic viability, yeasts need to adapt continuously to changing growth conditions and increased ethanol concentrations. Saccharomyces cerevisiae is favored industrially for bread making, brewing, wine making, and bioethanol production (4). Concerns regarding oil depletion, climate change, and, presently, the COVID-19 pandemic have spurred demands for bioethanol, the largest biotechnology product and most dominant biofuel, and for alcohol-based sanitizers and disinfectants. At present, metabolic engineering of yeast strains has become a necessary and effective strategy to ensure adequate large-scale production of bioethanol. However, maximizing ethanol yield remains a technical challenge. Comprehension of yeast responses to osmotic, oxidative, thermal, and ethanol stress during alcoholic fermentation and tolerance mechanisms is vital to improving fermentation efficiency.

The genome, proteome, and metabolome of yeast cells contain biomarkers of stress response. Several hundred genes are associated with ethanol sensitivity and tolerance and cover a broad range of functional categories, including cellular processes contributing to cell survival, such as the synthesis of proteins (heat shock proteins) and solutes, transport, and cell cycle and growth, as well as membrane and cell wall organization (5). Yeasts generally resist environmental stress, such as high ethanol concentrations, by modulation of trehalose, glycogen, and cell wall composition (6, 7). Ethanol induces the expression of genes related to trehalose and glycogen synthesis to facilitate a stable intracellular environment and maintain cell survival (8).

The molecular mechanism of yeast responses to environmental stress during alcoholic fermentation involves the activation of specific and general stress response programs. To protect itself and repair damages caused by stresses, the mitogen-activating protein kinases (MAPKs) of intracellular signaling play an important role. In S. cerevisiae, the high-osmolarity glycerol (HOG) pathway mediates the osmotic stress response, which is caused by high sugar concentrations or salt stress, by stimulating transcription regulators of glycerol biosynthesis, including the highly conserved Hog1 protein (9, 10). In addition, the transcriptional factors Msn2 and Msn4 control general stress-responsive genes containing the stress-responsive elements (STREs). They mediate kinase A-dependent gene expression and sensitivity to oxidative, thermal, and osmotic stresses (11, 12). Notably, expression rewiring during ethanol stress is under the control of transcription factors Msn2/4, Yap1, and Hsf1, which share binding motifs in upstream sequences of ethanol-tolerant gene targets (8). Increased levels of stress-protective proteins and the synthesis of defensive compounds or metabolites result in changes in cellular metabolism and structure following activation of the ethanol stress response (ESR). High sugar concentrations also lead to increased ethanol concentrations of up to 8 to 11% (vol/vol), which relatedly trigger a thermal stress response (13). The repression of protein biosynthesis and induction of genes that encode heat shock proteins (HSPs) are important protective mechanisms during thermal and ethanol stress because they function as molecular chaperones required for protection against damaged proteins. Some key heat shock proteins, such as Hsp104, are required for disaggregation of denatured proteins and play an additional role in yeast tolerance to ethanol (14, 15).

Among other transcription factors, Znf1 is a member of the major subfamily of zinc cluster DNA-binding proteins in S. cerevisiae (16, 17). Znf1 regulates *IMA* genes for isomaltulose sugar utilization (18). Additionally, Znf1 regulates many genes involved in the utilization of alternative carbon sources during the glucose-ethanol shift and is required for pH and osmotic stress responses (16). Moreover, Znf1 is a key transcription regulator of central carbon metabolism involved in controlling glycolysis and gluconeogenesis (16, 19). *ZNF1* overexpression enhances bioethanol production at high glucose concentrations and increases acetic acid tolerance (16, 19). Despite the role of Znf1 in bioethanol production and stress response, its involvement in ethanol stress adaptation is unknown. Close examination of Znf1 target gene regulation during the fermentation process may reveal important insights into metabolic regulation and cellular responses to increase ethanol stress tolerance. Genetic engineering, including CRISPR-Cas9, was employed to develop the ethanologenic *ZNF1*-overexpressing strain of S. cerevisiae for further improvement of cell survival during alcoholic fermentation.

## RESULTS

### Role of Znf1 in the biosynthesis of glycerol and fatty acids during ethanol stress.

**FIG 1:**
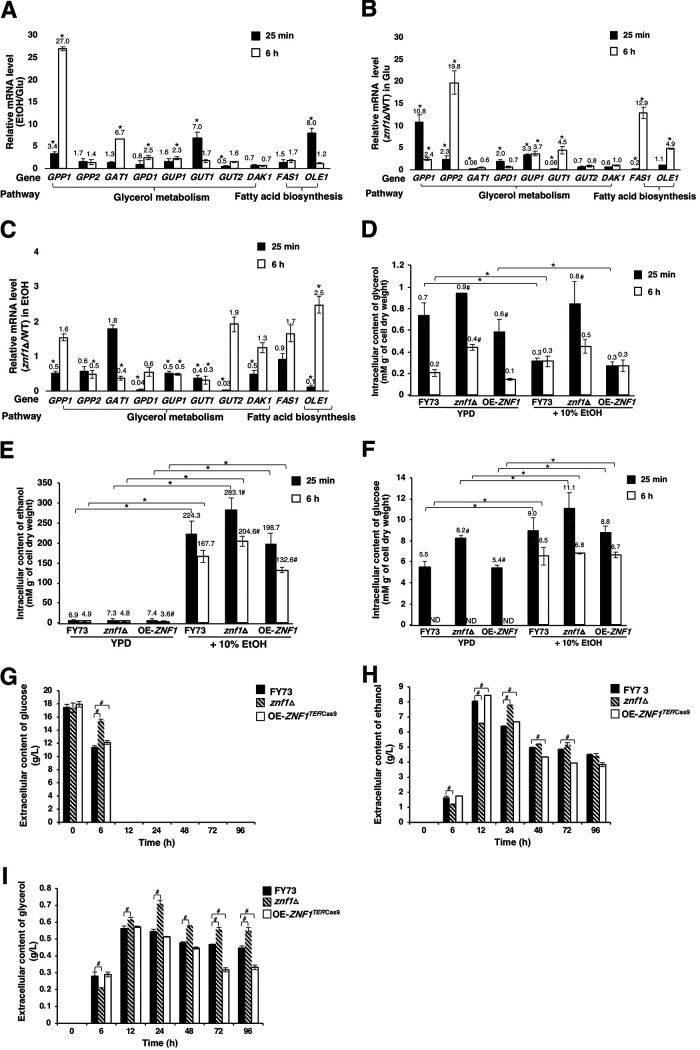
Znf1-regulated expression of genes involved in the metabolism of glycerol and fatty acids in response to ethanol (EtOH) stress. (A) Relative mRNA levels of some genes in the wild-type (FY73) S. cerevisiae strain grown at a 10% (vol/vol) ethanol concentration in glucose for 25 min or 6 h, versus in glucose only. (B) Relative expression levels of genes under normal glucose conditions and (C) under ethanol stress conditions in the *znf1*Δ and wild-type strains. Relative expression levels were obtained via the comparative threshold cycle (*C_T_*) method for quantification of 2^−ΔΔ^*^CT^* values. Relative expression of 2-fold lower or higher was considered significant (*). Error bars indicate standard deviations calculated from at least two independent experiments performed in triplicate. The wild-type (FY73), *znf1*Δ, and *ZNF1*-overexpressing (OE-*ZNF1^TEF^*^/Cas9^) strains cultured in YPD or treated with 10% (vol/vol) ethanol were analyzed for intracellular (D) glycerol, (E) ethanol, and (F) glucose contents and for extracellular (G) glucose, (H) ethanol, and (I) glycerol contents by high-performance liquid chromatography (HPLC). * and # indicate a *P* value of <0.05, by two-tailed Student’s *t*-test comparisons to the untreated condition and the wild-type strain, respectively.

Yeast cells employ various mechanisms of cellular protection to reprogram gene expression and synthesize necessary cellular components when exposed to stressful events. Glycerol is known to protect yeast cells from environmental stress during fermentation and is relevant to osmoadaptation via the HOG pathway (9, 20). Ethanol stress induces a decrease in the lipid content of the plasma membrane and alters the fluidity of the cell structure (21). Several hundred genes involving a broad range of functional categories, including the biosynthesis of glycerol and fatty acids, are associated with ethanol tolerance. Using quantitative real-time PCR (qRT-PCR), we investigated changes in the mRNA levels of glycerol and fatty acid biosynthetic genes in response to 10% (vol/vol) ethanol treatment in the wild-type and the *znf1*Δ strains of S. cerevisiae. In glucose, the relative mRNA levels of some genes were significantly upregulated, namely, those of *GPP1*, encoding glycerol-1-phosphatase; *GAT1*, involved in the triacylglycerol pathway; *GPD1*, encoding glycerol-3-phosphate dehydrogenase; *GUP1*, encoding glycerol uptake; and *OLE1*, required for monounsaturated fatty acid synthesis, indicating the function of glycerol and fatty acids in ethanol stress response (Fig. 1A). Znf1 also activated *GAT1* and *FAS1* expression in glucose (Fig. 1B). However, Znf1 repressed the expression of *GPP1*, *GUP1*, *GUT1*, *FAS1*, and *OLE1* in glucose (Fig. 1B).

In response to the 10% (vol/vol) ethanol treatment, Znf1 activated the expression of many genes, including that of *GPP1* and *DAK1* during the early response and that of *GPP2*, *GAT1*, *GUP1*, and *GUT1* at a later time point (6 h) (Fig. 1C). Under ethanol stress conditions, expression of *GCY1* (encoding glycerol dehydrogenase), *GPD1* (glycerol-3-phosphate dehydrogenase), and *DAK1* (dihydroxyacetone kinase) genes, involved in glycerol metabolism, are upregulated in ethanol-tolerant yeast strains (5). The glycerol uptake 1 (*GUP1*) gene encodes glycerol uptake transporters implicated in a wide range of processes relating to cell preservation, including membrane and cell wall composition and lipid metabolism, in terms of sphingolipids and sterol domain integrity and assembly (22, 23). Overexpression of the *GUP1* gene in yeast induces proliferation of intracellular membranes containing endoplasmic reticulum (ER), Golgi, and itinerant proteins (24). Ethanol stress-responsive genes linked to lipid and phospholipid metabolism and the regulation of lipids and fatty acids have also been identified (25, 26). The *znf1*Δ strain showed significantly decreased expression of the fatty acid synthetase *FAS1* and increased expression of the delta-9 fatty acid desaturase *OLE1* at 25 min and 6 h, respectively, compared to that in the wild-type strain. Thus, the results indicated an activator and a repressor function of Znf1 in mediating fatty acid biosynthesis (Fig. 1C).

### Dynamic regulation of glycerol production by Znf1 and its role in plasma membrane protection.

The glycerol, ethanol, and glucose contents of the S. cerevisiae strains were also analyzed via high-performance liquid chromatography (HPLC). After 25 min of the ethanol treatment, the wild-type, *znf1*Δ, and *ZNF1*-overexpressing strains displayed intracellular glycerol accumulation of 0.7, 0.9, and 0.6 mM/g cell dry weight, respectively, in glucose, which decreased to 0.2, 0.4, and 0.1 mM/g cell dry weight after 6 h (Fig. 1D). The decrease in glycerol contents of all tested strains suggested the utilization of the accumulated glycerol. In glucose, Znf1 blocked the production of glycerol but activated its utilization. As shown, the *ZNF1*-overexpressing strain quickly utilized the accumulated glycerol in glucose-containing medium (Fig. 1D). However, the reverse effect was observed after treatment with ethanol at 6 h. The glycerol content was dramatically reduced as glycerol was converted to glucose or other metabolites (Fig. 1D). The intracellular glycerol content was significantly lower in the wild-type and overexpression strains compared to that in the *znf1*Δ strain, suggesting Znf1-dependent conversion of glucose-ethanol mix to glycerol (Fig. 1D). No significant change in the glycerol content between the wild-type and the *ZNF1*-overexpressing strains was found (Fig. 1D), suggesting that glycerol biosynthesis and glycerol utilization occur simultaneously in the presence of high ethanol concentration. This agreed well with the Znf1-dependent ethanol induction of *GPP1* and *GPD1* expression for glycerol biosynthesis and the subsequent increase in *GUT1*, *GUT2*, and *DAK1* expression for glycerol utilization (Fig. 1A to C). Noticeably, glycerol contents of the wild-type and *ZNF1-*overexpressing strains remained constant during the 6-h treatment with ethanol, suggesting the simultaneous production and utilization of glycerol by Znf1 during the adaptive response to ethanol stress (Fig. 1D).

Glucose to ethanol conversion was compromised in the *znf1*Δ strain, as expected, since the Znf1 transcription factor is involved in the induction of genes in the lower pathway of glycolysis (19). Additionally, deletion of *ZNF1* allowed for glycerol production from glucose through derepression of *GPD1* and *GPP1/2* expression (Fig. 1B). After glucose depletion, utilization of glycerol and activation of gluconeogenesis for glucose-ethanol conversion is also compromised in the *znf1*Δ strain (16). Therefore, glycerol was increasingly accumulated, and glucose was then converted to glycerol in the *znf1*Δ strain, as shown in Fig. 1D.

In contrast, the intracellular ethanol content was very low in glucose-grown cells, approximately 3.6 to 7.4 mM/g of cell dry weight, but massively increased after ethanol treatment, as expected for all strains, by approximately 30- to 40-fold (Fig. 1E). The highest intracellular ethanol level was found in the *znf1*Δ strain due to defective utilization of ethanol, as previously shown (Fig. 1E) (16). Furthermore, the *ZNF1*-overexpressing strain displayed the lowest intracellular ethanol content, which is likely due to the release of ethanol produced (Fig. 1H). As expected, the intracellular glucose level was quite high in glucose-grown cells (Fig. 1F), especially in the *znf1*Δ strain lacking a transcription regulator of glycolysis (19).

Interestingly, treatment with ethanol in glucose-grown cells resulted in increased intracellular glucose levels for all tested strains, suggesting activation of gluconeogenesis for the ethanol-glucose conversion (Fig. 1F). The highest intracellular glucose content was found in the *znf1*Δ strain (Fig. 1F) due to deletion of the key gluconeogenic regulator Znf1 (16). Apparently, after 6 h, the intracellular glucose content of cells grown in glucose culture was not detectable due to complete glucose consumption in all tested strains (Fig. 1F). However, increased intracellular glucose contents were found in cells grown in the mixed glucose-ethanol culture after 25 min of treatment (Fig. 1F). The synthesized glucose after 6 h of ethanol treatment may be from gluconeogenesis. Due to impaired gluconeogenesis, the glucose content was higher in the *znf1*Δ strain compared to those in the wild-type and *ZNF1-*overexpressing strains (Fig. 1F).

The extracellular glucose, glycerol, and ethanol concentrations were also examined. Glucose consumption was rapidly completed within 12 h of treatment for all strains; a lower rate of glucose consumption was found in the *znf1*Δ strain compared to that in the wild-type strain (Fig. 1G). The *znf1*Δ strain exhibited lower ethanol production (6.58 g/liter) than that of the wild-type strain (8.06 g/liter; Fig. 1H). However, the *ZNF1*-overexpressing strain exhibited high ethanol production (8.43 g/liter) and low glycerol production (Fig. 1H and I). After 12 h and following complete glucose consumption, the *ZNF1*-overexpressing strain significantly utilized ethanol and glycerol as alternative carbon sources (Fig. 1G to I). After 12 h, the *znf1*Δ strain showed a significantly higher level of glycerol production (0.61 g/liter) than that of the wild-type strain (0.55 g/liter), an increase of 10.9% (Fig. 1G and I). In comparison, the *znf1*Δ mutant was slower in utilizing the alternative carbon sources. As shown, Znf1 also activated the expression of glycerol catabolic genes, such as *GAT1* and *GUT1* for the synthesis of triacylglycerol, which are a key component of fatty acids present in the plasma membrane (Fig. 1C).

**FIG 2:**
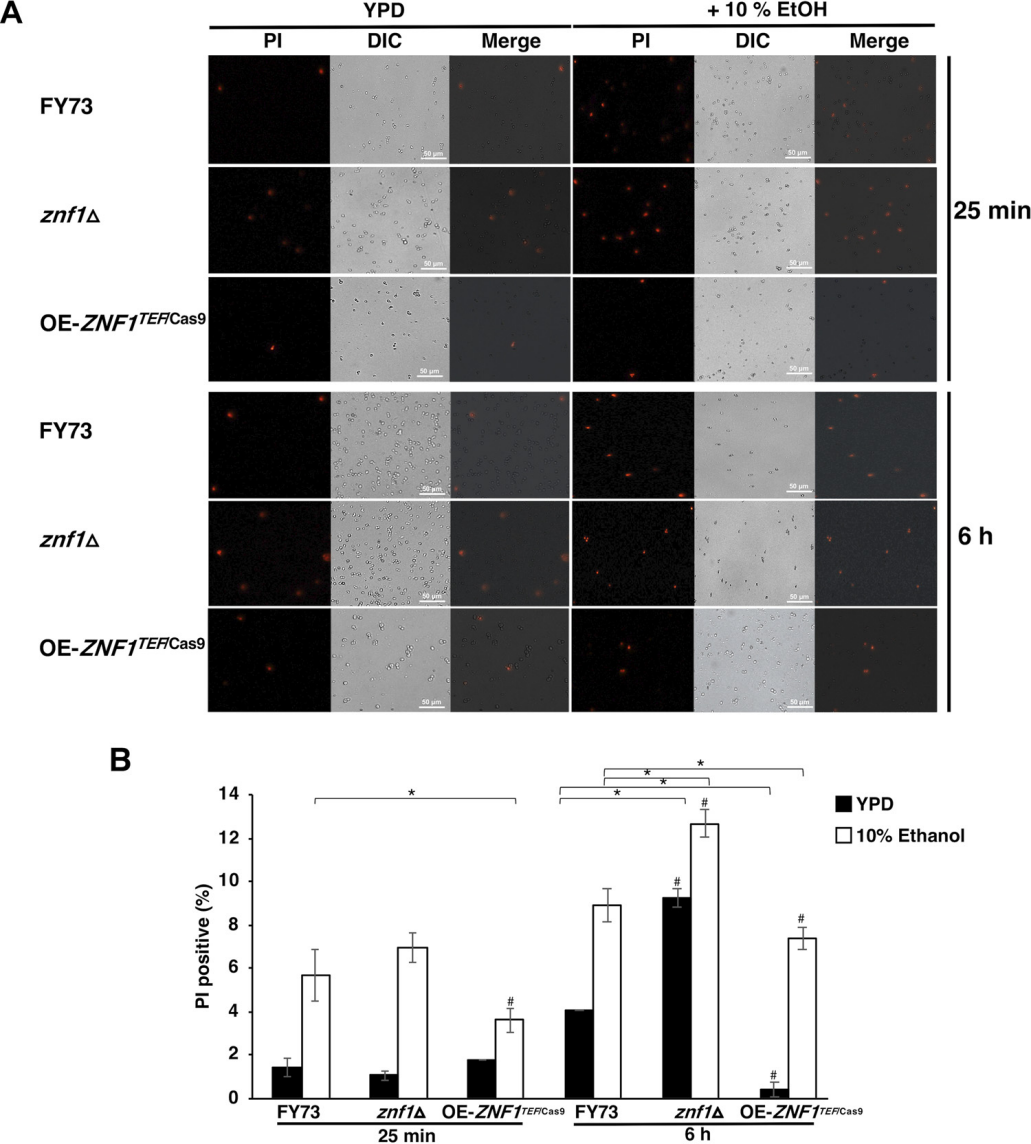
Effect of ethanol treatment on cell membrane integrity. The wild-type, *znf1*Δ, and *ZNF1*-overexpressing strains were treated with ethanol at different time points (25 min and 6 h) to examine the cell membrane integrity (A) and the plasma membrane integrity (propidium iodide [PI]) assay (B). The percentage of PI-positive cells of yeast strains was quantified using at least two independent experiments conducted in triplicate.

To investigate plasma membrane integrity, propidium iodide (PI) stain, a fluorescent intercalating agent that binds to the nucleic acid of cells, was applied following the ethanol treatments. The plasma membrane integrity was compromised, as shown by PI staining, after ethanol treatment compared to that in untreated cells. The *znf1*Δ strain showed 6.9% PI positives compared to 5.6% PI positives in the wild-type strain at 25 min in the presence of 10% (vol/vol) ethanol, with 12.7% and 8.9% PI positives exhibited at 6 h, respectively. The *ZNF1*-overexpressing strain showed a less defective plasma membrane, with 3.6% PI positives at 25 min and 7.4% PI positives at 6 h (Fig. 2), suggesting the involvement of *ZNF1* in resisting ethanol stress.

### Znf1-modulated carbohydrate metabolism during ethanol stress.

Yeasts produce a variety of metabolites that are altered in the presence of increasing ethanol concentrations (27). These include trehalose as a nonreducing disaccharide of glucose, glycogen as a polysaccharide of glucose, and mannan as a cell wall polysaccharide composed of mannose units (28). Carbohydrate storage from the trehalose biosynthesis pathway could be subtly affected by biosynthesis and catabolism of glycogen, which is involved in the biosynthesis and function of cell wall components such as glucan molecules and the reserve energy of cells. S. cerevisiae responds to ethanol stress conditions by reprogramming the expression of genes in carbohydrate metabolism for biosynthesis of trehalose, glycogen, and cell wall components (29).

**FIG 3:**
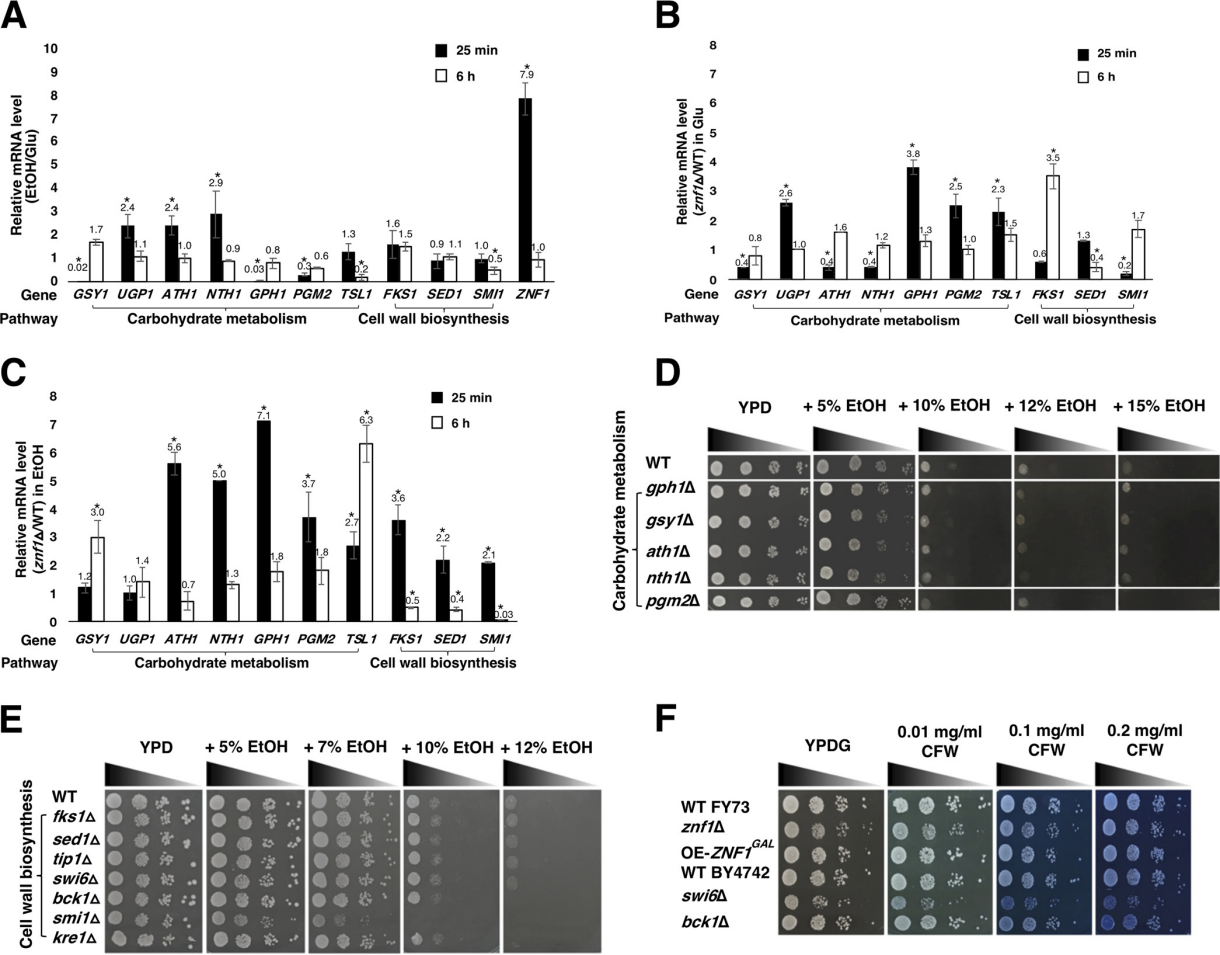
Role of Znf1 in controlling carbohydrate metabolism and cell wall biosynthesis during the ethanol stress response (ESR). (A) Relative expression levels of stress-responsive genes in the wild-type (FY73) S. cerevisiae strain grown in glucose-containing medium with 10% (vol/vol) ethanol for 25 min and 6 h, versus with glucose only. (B) Relative expression levels of genes under normal glucose conditions and (C) ethanol stress conditions in the *znf1*Δ and wild-type strains. Relative expression levels were obtained via the comparative *C_T_* method for quantification of 2^−ΔΔ^*^CT^* values. Relative expression of 2-fold lower or higher was considered significant (*). Error bars indicate standard deviations calculated from at least two independent experiments performed in triplicate. (D and E) Phenotypes of the FY73 and BY4742 (wild-type) strains and strains lacking genes encoding carbohydrate and cell wall pathways. These strains were incubated on YPD solid medium supplemented with 5, 7, 10, 12, or 15% (vol/vol) ethanol. Cells were serially diluted 10-fold from 10^−1^ to 10^−4^, with an initial optical density at 600 nm (OD_600_) of 0.1 and incubated at 30°C for 2 to 3 days. (F) Phenotypes of yeast strains in the presence of cell wall stress. FY73 and BY4742 wild-type (WT), *ZNF1*-overexpressing pYES2-*ZNF1*(OE*-ZNF1^GAL^*), and *znf1*Δ strains were spotted on YPDG solid medium supplemented with 0.01, 0.1, or 0.2 mg/ml calcofluor white (CFW). Cells were serially diluted 10-fold from 10^−1^ to 10^−4^ with an initial OD_600_ of 0.1 and incubated at 30°C for 48 h. The experiment was performed in triplicate.

Here, the wild-type and *znf1*Δ strains were treated with high ethanol concentrations for 25 min and 6 h to examine the expression of genes associated with carbohydrate metabolism. Gene expression studies showed that the mRNA levels of *GSY1* (6 h), *UGP1*, *ATH1*, and *NTH1* (25 min) increased by 2- to 3-fold relative to those of the wild-type strain, in response to the 10% (vol/vol) ethanol (Fig. 3A). *GSY1* encodes glycogen synthase, which is expressed under glucose-limiting conditions and during environmental stress. *UGP1* encodes UDP-glucose pyrophosphorylase, which is involved in numerous metabolic pathways. *ATH1* and *NTH1* are relevant for trehalose degradation and are required for cellular stress response. Regarding the function of transcription factor Znf1, at 25 min, 2- to 5-fold upregulation of *UGP1*, *GPH1*, *PGM2*, and *TSL1* and downregulation of *GSY1*, *ATH1*, *NTH1*, and *SMI1* compared to expression in the wild-type strain were found in the glucose-grown *znf1*Δ strain (Fig. 3B). This suggests that, at the early stage of fermentation, Znf1 represses trehalose biosynthesis and activates glycogen biosynthesis and trehalose degradation, leading to cell wall biosynthesis (Fig. 3B). Previous studies have reported high neutral trehalase activity of Nth1p in yeast cells at an early growth stage and that it is inactivated at the stationary phase of growth (30, 31).

Accumulation of trehalose is reflected by upregulation of genes of trehalose synthesis under ethanol stress (8). Overexpression of *TPS1* and deletion of *NTH1* genes have been reported to enhance ethanol stress tolerance in yeast by increasing the accumulation of trehalose (6). Simultaneously induced expression of genes involved in glycogen biosynthesis and degradation, namely *GSY1* and *GSY2* (glycogen synthase) and *GPH1* (glycogen phosphorylase) have been observed under ethanol stress (8). At the 10% (vol/vol) ethanol treatment, upregulation of *GSY1*, encoding glycogen synthase (3.0-fold), and *TSL1*, encoding trehalose synthase (6.3-fold), was found in the absence of Znf1, indicating the repressive role of Znf1 (Fig. 3C). However, Znf1 upregulated the expression of *FKS1*, encoding a catalytic subunit of 1,3-β-d-glucan synthase, which involved in cell wall synthesis, maintenance, and remodeling, by 2.0-fold, *SED1* encoding a major stress-induced structural glycosylphosphatidylinositol (GPI)-cell wall glycoprotein associated with translating ribosomes by 2.3-fold and strongly induced the expression of *SMI1*, encoding a regulator of cell wall biosynthesis, by 33.3-fold (Fig. 3C). The activation of *FKS1*, *SED1*, and *SMI1* genes by Znf1 may enhance cell wall stability during ethanol stress. However, Znf1 repressed the expression of genes involved in carbohydrate metabolism and cell wall biosynthesis (Fig. 3C). Thus, cell wall stability represents a key mechanism to ESR.

In addition, spot assays of the single-deletion (of genes related to carbohydrate metabolism and cell wall biosynthesis) strains were also performed at 5, 10, 12, and 15% (vol/vol) ethanol concentrations. The *gph1*Δ, *gsy1*Δ, *ath1*Δ, *nth1*Δ, and *pgm2*Δ strains involved in trehalose or glycogen biosynthesis showed slightly impaired growth compared to that of the wild-type strain under ethanol stress (Fig. 3D). The deletion of genes encoding cell wall components, such as *SMI1*, *BCK1*, and *KRE1*, resulted in impaired growth at high ethanol concentrations compared to that of the wild-type strain (Fig. 3E). Importantly, these genes were Znf1 target genes. *SMI1* encodes a protein involved in the regulation of cell wall synthesis (32); *BCK1* encodes mitogen-activated protein kinase (MAPK) that acts in the protein kinase C signaling pathway, which controls cell integrity (33); and *KRE1* encodes a cell wall glycoprotein involved in β-glucan assembly (34). Finally, we also examined the effect of cell wall stress on the growth of mutant strains in the presence of 0.01, 0.1, or 0.2 mg/ml calcofluor white (CFW), a cell wall-perturbing agent that binds to chitin. The wild-type and *ZNF1*-overexpressing strains grew normally in the presence of ethanol and CFW, while the *znf1*Δ strain and, more evidently, the *swi6*Δ strain showed slightly impaired growth (Fig. 3F). The results also showed that the *znf1*Δ and *swi6*Δ deletion strains had impaired growth both in yeast extract-peptone-dextose-galactose (YPDG) and ethanol-containing media, suggesting that these genes are not specifically required for ethanol stress adaptation. They may also play roles in other cellular processes. Thus, the Znf1 transcription factor appears to regulate expression of some key genes associated with carbohydrate metabolism and maintenance of cell wall integrity during ethanol and cell wall stresses.

### High glycerol production in strains lacking genes associated with cell wall biosynthesis.

**FIG 4:**
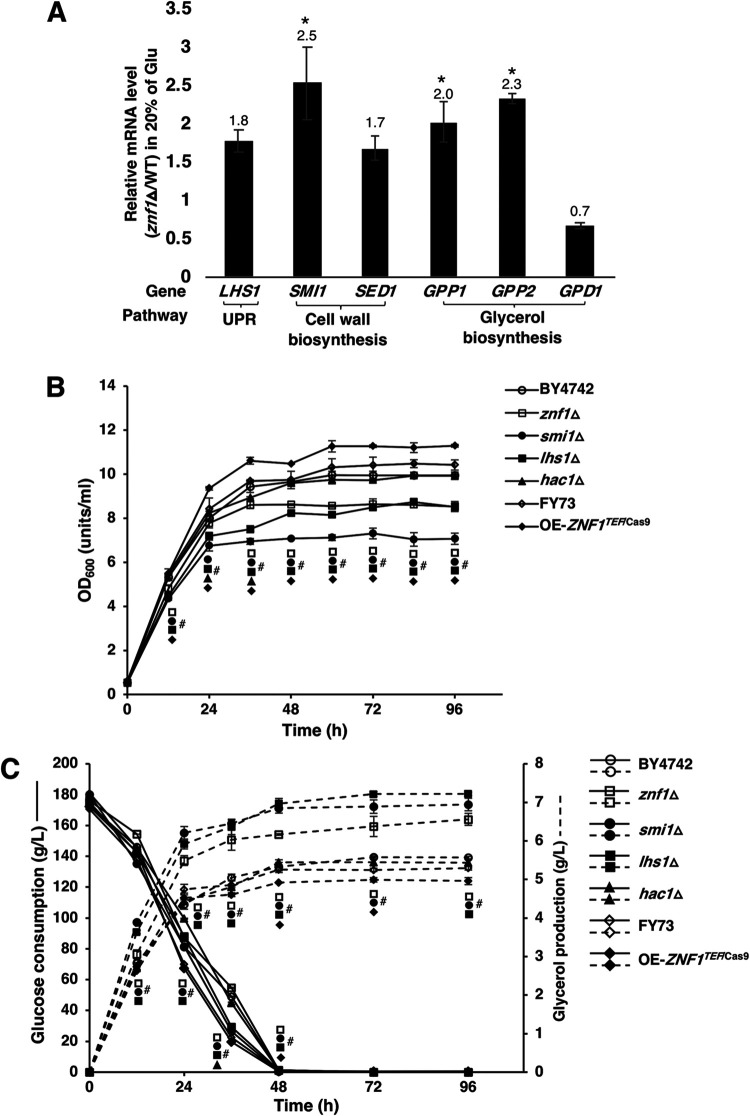
Glycerol-induced fermentation profiles in yeast Saccharomyces cerevisiae. The wild-type (FY73 and BY4742), *znf1*Δ, *smi1*Δ, *hac1*Δ, *lhs1*Δ, and *ZNF1*-overexpressing (OE-*ZNF1^TEF^*^/Cas9^) strains were grown with 20% (wt/vol) glucose as a substrate. (A) Relative expression levels of ethanol stress-responsive genes under high-glucose (20% wt/vol) conditions. Relative expression levels were obtained via the comparative *C_T_* method for quantification of 2^−ΔΔ^*^CT^* values. Relative expression of 2-fold lower or higher was considered significant (*). Error bars indicate standard deviations calculated from at least two independent experiments performed in triplicate. (B) Cells were cultured at 20% (wt/vol) glucose for 12 to 96 h, and cell growth was determined by OD_600_ using a spectrophotometer for indicated strains. (C) Glucose consumption and glycerol production of the wild-type, *znf1*Δ, *smi1*Δ, *hac1*Δ, and *lhs1*Δ strains grown with 20% (wt/vol) glucose for 96 h. Average values of the fermentation profiles were calculated from two independent experiments conducted in triplicate. #, *P* < 0.05, two-tailed Student’s *t* test compared to the wild-type strain.

The cell wall of yeasts contains mannoproteins (35 to 40%), β-glucan (30 to 60%), and chitin (1 to 2%) (35). Upregulated expression of genes related to the cell wall structure in response to ethanol stress, including *TIP1*, which is linked to mannoprotein metabolism, has been reported (8, 25). Most genes involved in cell membrane and cell wall contents are downregulated, and the membrane and cell wall structures undergo significant remodeling processes to attain homeostasis in response to ethanol stress (36). Our results also showed that, at 24 h of 20% (wt/vol) glucose fermentation, the *znf1*Δ strain showed upregulation of *SMI1*, *GPP1*, and *GPP2*, resulting in increased glycerol production (Fig. 4A). Additionally, the *smi1*Δ, *lhs1*Δ, and *znf1*Δ deletion strains also showed poor growth compared to that of the wild-type strain (Fig. 4B), indicating their importance in growth maintenance during ethanol stress. As a key target of Znf1, *SMI1* regulates cell wall biosynthesis and integrity. Its deletion leads to significant growth reduction under high temperature or CFW stress (37, 38). Also, the *smi1*Δ strain displayed increased release of polysaccharides and mannoproteins into the culture medium, which is of special interest for certain fermentation processes (39). Cell wall biosynthesis is also associated with the cell wall integrity signaling HOG pathway (9, 40). In fact, *SMI1* deletion not only results in defective cell wall structure but also increases autolysis during fermentation and decreases cell proliferation (41). Additionally, *LHS1* expression is upregulated under ethanol stress in the tolerant yeast strain CECT10094 (15). Deletion of the *LHS1* gene, which is involved in ATP binding, also leads to a low ATP level and decreased cell survival in the presence of proteotoxicity (42). Growth comparisons with the wild-type strain revealed that the overexpression strain OE-*ZNF1^TEF^*^/Cas9^ showed the best growth during fermentation, while the *smi1*Δ strain grew most poorly (Fig. 4B). For fermentation, glucose consumption was nearly completed after 48 h for all strains tested (Fig. 4C). Cells responded to high ethanol levels by producing more glycerol during fermentation, especially those of the *znf1*Δ strain (Fig. 4C). The *lsh1*Δ, *smi1*Δ, and *znf1*Δ deletion strains showed increased glycerol production of 7.21, 6.85, and 6.17 g/liter, respectively, compared to 5.3 g/liter by the wild-type strain (Fig. 4C). Probably, deletion of *SMI1,* a key target of Znf1 that is involved in coordinating cell cycle progression and cell wall integrity, may have various effects, particularly during stress. Thus, Znf1 regulation of cell wall signaling and biosynthesis is linked to key metabolic pathways, including glycerol and fatty acid metabolisms.

### Znf1-regulated expression of genes in the unfolded protein response pathway.

**FIG 5:**
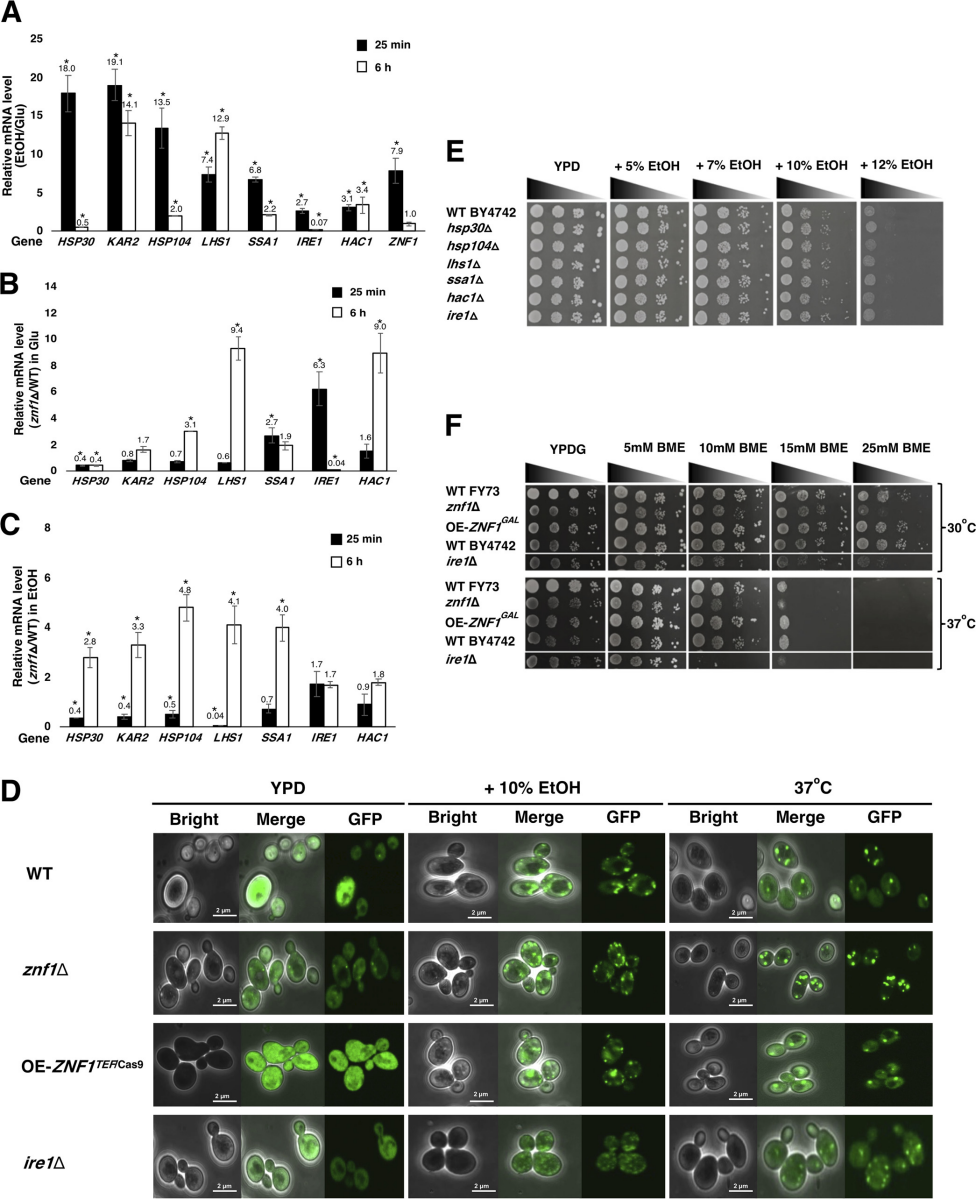
Znf1 activated the unfolded protein response (UPR) during the ethanol stress. (A) Relative mRNA levels of UPR genes in the wild-type (FY73) S. cerevisiae strain grown in glucose-containing medium with 10% (vol/vol) ethanol for 25 min or 6 h, versus with medium containing glucose only. Relative expression levels of UPR genes in glucose (B) and during ethanol stress (C) in the *znf1*Δ strain versus the wild-type strain at 25 min or 6 h. Relative expression levels were obtained via the comparative *C_T_* method for quantification of 2^−ΔΔ^*^CT^* values. Relative expression of 2-fold lower or higher was considered significant (*). Error bars indicate standard deviations calculated from at least two independent experiments performed in triplicate. (D) Hsp104-GFP expression in yeast under ethanol stress. Cells were grown at 30°C for 25 min in YPD with 10% (vol/vol) ethanol until an OD_600_ of 0.6 or at 37^°^C with no ethanol treatment. Localization of Hsp104-GFP and the formation of Hsp104-GFP foci were analyzed by confocal microscopy. (E) Phenotypes of the wild-type and deletion strains lacking genes in the UPR pathway during ethanol stress. These strains were grown on YPD solid medium supplemented with 5, 7, 10, and 12% (vol/vol) ethanol. The cells were serially diluted 10-fold from 10^−1^ to 10^−4^ with an initial OD_600_ of 0.1 and incubated at 30°C for 3 days. (F) Phenotypes of yeast strains in the presence of the protein folding stressor β-mercaptoethanol (BME). Wild-type (FY73 and BY4742), *ZNF1-*overexpressing (OE-*ZNF1^GAL^*), and *znf1*Δ strains were spotted on YPDG solid medium supplemented with 5, 10, 15, or 25 mM BME, and cells were serially diluted 10-fold from 10^−1^ to 10^−4^, with an initial OD_600_ of 0.1, and incubated at 30°C and 37°C for 3 days.

Cellular responses to stress and the repair protein functions are mediated by a core set of heat shock proteins (HSPs). Ethanol stress induces the activation of genes for cellular protection and survival, including those encoding the unfolded protein response (UPR) pathway (15). The UPR functions to stabilize membranes and proteins and inhibit protein aggregation during refolding, and effectively protect yeast cells against ethanol stress (43). Gene expression studies were used to investigate the involvement of the transcription factor Znf1 in mediating UPR genes during the ethanol stress response. At 10% (vol/vol) ethanol concentration, the relative mRNA levels of UPR genes were upregulated in the wild-type strain at an early time point (25 min) of the ethanol treatment (Fig. 5A). The ethanol-induced genes included *HSP30* (18.0-fold), linked to stress-responsive proteins; *KAR2*, encoding ATPase involved in protein import into the ER; *HSP104*, involved in protein folding; *LHS1*, associated with molecular chaperones in the ER lumen; *SSA1*, encoding ATPase involved in protein folding; *HAC1*, encoding transcription factors of the UPR; *IRE1*, a signal for spliced *HAC1* mRNA regulation of UPR genes; and *ZNF1*, involved in glycolysis and gluconeogenesis (Fig. 5A). During a late response phase, at 6 h following the ethanol treatment, some genes, including *KAR2*, *LHS1*, *SSA1*, and *HAC1* remained activated, but at lower levels, suggesting an early response of these genes (Fig. 5A). The mRNA levels of some genes, including those of *HSP30*, *HSP104*, and *IRE1*, were either unchanged or repressed, suggesting a quick response to ethanol that declined over time (Fig. 5A).

The role of Znf1 in the transcriptional regulation of UPR stress-responsive genes was also studied via qRT-PCR. The relative expression of genes in the *znf1*Δ and wild-type strains under glucose and ethanol treatments were investigated. The relative mRNA level of *HSP30* was significantly downregulated in glucose-treated *znf1*Δ strain by 2-fold, indicating an activator role of Znf1 (Fig. 5B). At 25 min, the mRNA levels of other UPR genes remained unaltered upon *ZNF1* deletion, except for those of *SSA1,* which was repressed by Znf1 (Fig. 5B). However, at 6 h, the mRNA levels of *HSP104*, *LHS1*, *SSA1*, *HAC1*, and *SSA1* were upregulated by 2- to 9-fold, indicating Znf1-dependent repression (Fig. 5B). With the 10% (vol/vol) ethanol treatment, the relative mRNA levels of *HSP30*, *KAR2*, *HSP104*, and *LHS1* were significantly downregulated in the absence of Znf1 at 25 min, suggesting early activation by regulator Znf1 (Fig. 5C). However, during late response at 6 h, Znf1 repressed the expression of *HSP30, KAR2*, *HSP104*, *LHS1*, and *SSA1* (Fig. 5C), suggesting a dual and dynamic role of Znf1 in mediating UPR regulation. The mRNA levels of the UPR regulatory transcription factors *HAC1* and *IRE1* remained unchanged in the *znf1*Δ strain (Fig. 5C), possibly suggesting a divergent control of the UPR pathway.

High ethanol concentrations negatively perturb protein conformation and cause the accumulation of denatured proteins, which can be monitored by the marker protein Hsp104p-green fluorescent protein (GFP) (44). Hsp104 is a key heat shock chaperone in the UPR system that repairs protein aggregation or misfolded proteins by binding to aggregated proteins and inducing UPR response to prevent disruption of cellular protein homeostasis (15). Next, the accumulation of protein aggregates in yeast cells under ethanol treatment and heat stress (control) was monitored by confocal analysis of Hsp104-GFP-targeted protein aggregates of the wild-type and *znf1*Δ strains. Disruption of the *IRE1* gene involved in sensing of the unfolded protein response (UPR) resulted in increased expression of Hsp104-GFP, indicating the accumulation of protein aggregates in the yeast cells (Fig. 5D). Importantly, as shown for the positive-control *ire1*Δ strain, the *znf1*Δ strain displayed a strong Hsp104-GFP signal, indicating high levels of protein aggregation at 10% (vol/vol) ethanol and 37°C compared to those in the wild-type strain (Fig. 5D).

Furthermore, the wild-type and deletion strain lacking the UPR gene were also spotted on yeast extract-peptone-dextrose (YPD) plates containing different ethanol concentrations (5 to12% [vol/vol]). A reduction in cell growth was observed with increasing ethanol concentrations for the wild-type and deletion strains (Fig. 5E). Similarly, cell survival decreased with increasing β-mercaptoethanol (BME) concentrations (Fig. 5F). Like ethanol, BME also affected the function of enzymes in the UPR pathway through disruption of protein folding by breaking disulfide bonds. High temperatures also denature proteins. The BME treatment was evaluated at 30°C and 37°C; the wild-type and *ZNF1-*overexpressing strains grew better than the *znf1*Δ strain and the UPR-defective *ire1*Δ strain, suggesting a role of Znf1 in mediating UPR response to these stress agents (Fig. 5E and F). The ethanol-tolerant CECT10094 strain induces activation of genes related to UPR and ER chaperones, such as *LHS1* and *KAR2* genes (15). These genes encode redox proteins and the corresponding targets of transcription factors Hac1 and Znf1. Overall, the results indicated an important and newly identified function of transcription factor Znf1 in regulating UPR gene expression and cell survival under ER and ethanol stress.

### The fermentation profiles of the *ZNF1* overexpression and *ZNF1*-*HSP104* coexpression strains.

**FIG 6:**
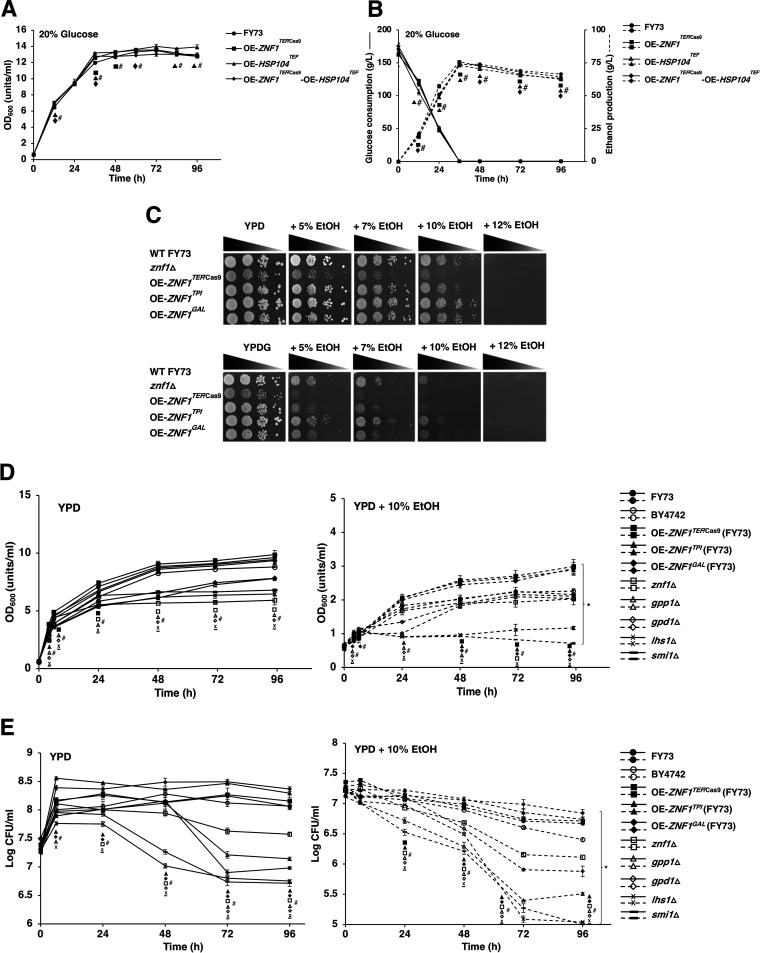
Fermentation profiles and effect of ethanol stress on yeast S. cerevisiae wild-type and engineered strains. The wild-type (FY73), *ZNF1* overexpression (OE*-ZNF1^TEF^*^/Cas9^), *HSP104* overexpression (OE*-HSP104^TEF^*), and *ZNF1*-*HSP104* co-overexpression (OE*-ZNF1^TEF^*^/Cas9^*-HSP104^TEF^*) strains were cultured with 20% (wt/vol) glucose for 12 to 96 h. (A) Cell growth as measured by the optical density (OD_600_) was determined by spectrophotometer, and (B) glucose consumption and ethanol production were determined by HPLC; data were based on two independent experiments conducted in triplicate. (C) Phenotypic analysis, (D) growth curve of yeast strains, and (E) survival of yeast strains under ethanol stress. The S. cerevisiae wild-type (FY73 and BY4742), *znf1* deletion (*znf1*Δ), *ZNF1*-overexpressing (OE-*ZNF1*), and deletion mutant (*gpp1*Δ, *gpd1*Δ, *lhs1*Δ, and *smi1*Δ) strains were spotted on YPD or YPDG solid medium supplemented with 5, 7, 10, or 12% (vol/vol) ethanol. Error bars indicate standard deviations calculated from at least two independent experiments performed in triplicate. * and #, *P* < 0.05 two-tailed Student’s *t* test compared to the untreated condition and the wild type, respectively.

Hsp104 is known to facilitate cell survival and adaptive responses to ethanol stress, but its role in ethanol production is unclear. Thus, the fermentation profiles of the wild-type, *znf1*Δ, *ZNF1-*overexpressing, *HSP104-*overexpressing, and *ZNF1–HSP104*-co-overexpressing strains were examined for ethanol production, using 20% (wt/vol) glucose as a substrate. As expected, the *ZNF1–HSP104*-co-overexpressing strain performed better than the rest, especially at 36 h, while the growth of strain *znf1*Δ was impaired the most (Fig. 6A). Our results showed that *HSP104* overexpression promotes cell biomass, while co-overexpression of *ZNF1* and *HSP104* increased cell biomass more than that in either the *HSP104*- or the *ZNF1*-overexpressing strains (Fig. 6A and Table 1), although the increase in biomass leveled off after 48 h for all strains, including the wild-type strain.

**TABLE 1:** Fermentation and biomass profiles of S. cerevisiae wild-type, *ZNF1* or *HSP104* overexpression, *ZNF1*-*HSP104* co-overexpression, and deletion strains

Parameter	Strain^*b*^									
	FY73	OE-*ZNF1^TEF^*^/Cas9^ (FY73)	OE-*HSP104^TEF^* (FY73)	OE-*ZNF1^TEF^*^/Cas9^-*HSP104^TEF^* (FY73)	*znf1*Δ (FY73)	BY4742	*znf1*Δ (BY4742)	*smi1*Δ (BY4742)	*lhs1*Δ (BY4742)	*hac1*Δ (BY4742)
Glucose consumption (g/liter · h^−1^)	4.72	4.49	4.62	4.86	4.55	3.45	3.37	4.27	4.04	3.78
	3.54	3.37	3.47	3.64	3.42	3.58	3.64	3.74	3.75	3.62
Glucose (g/liter)	0.64 ± 0.02	0.60 ± 0.03	0.52 ± 0.02	0.44 ± 0.06	0.54 ± 0.14	48.98 ± 1.00	54.67 ± 2.1	26.45 ± 0.94	29.34 ± 1.57	44.76 ± 2.40
	0.65 ± 0.03	0.61 ±0.06	0.53 ± 0.03	0.49 ± 0.02	0.00 ± 0.00	0.96 ± 0.18	1.18 ±0.09	0.50 ± 0.09	1.22 ± 0.27	1.11 ± 0.03
Glycerol concentration (g/liter)	4.55 ± 0.13	4.39 ±0.38	4.55 ± 0.09	4.53 ± 0.01	6.23 ± 0.14	5.05 ± 0.08	6.04 ± 0.27	6.45 ± 0.08	6.36 ± 0.19	4.80 ± 0.02
	4.51 ± 0.07	4.82 ±0.01	4.68 ± 0.13	4.65 ± 0.04	6.30 ± 0.17	5.33 ± 0.10	6.12 ± 0.02	6.85 ± 0.12	6.97 ± 0.13	5.44 ± 0.06
Change in glycerol concentration (%)		−3.52	0	−0.44%	+36.92		+19.60	+27.72	+25.94	−4.95
		+6.87	+3.77	+3.10	+39.69		+14.82	+28.52	+30.77	+2.06
Glycerol yield (g per g of consumed glucose)	0.027	0.027	0.027	0.026	0.038	0.041	0.05	0.042	0.044	0.035
	0.027	0.03	0.028	0.027	0.038	0.031	0.035	0.038	0.04	0.03
Glycerol productivity (g/liter · h^−1^)	0.126	0.122	0.126	0.126	0.173	0.14	0.178	0.179	0.177	0.133
	0.094	0.1	0.098	0.097	0.131	0.111	0.128	0.143	0.145	0.113
Glycerol productivity (g/liter · g^−1^ of biomass/h)	0.046	0.044	0.044	0.042	0.067	0.067	0.086	0.087	0.088	0.06
	0.035	0.038	0.037	0.034	0.052	0.05	0.065	0.69	0.069	0.05
Ethanol concentration (g/liter)	73.71 ± 0.51	75.78 ± 0.08	73.09 ± 0.31	74.34 ± 0.56	71.58 ± 0.55	70.62 ± 0.28	68.59 ± 0.27	73.82 ± 0.48	68.08 ± 0.43	72.43 ± 0.42
	74.00 ± 0.31	72.88 ± 0.07	70.45 ±0.86	72.74 ± 0.33	77.56 ± 0.07	73.41 ± 0.12	69.34 ± 0.02	71.09 ± 0.14	64.95 ± 0.19	71.54 ± 0.20
Change in ethanol concentration (%)		+2.80	−0.84	+0.86	−2.89		−2.87	+4.53	−3.60	+2.56
		−1.51	−4.80	−1.70	+4.81		−5.54	−3.16	−12.02	−2.55
Ethanol yield (g per g of consumed glucose)	0.43	0.47	0.44	0.42	0.44	0.57	0.57	0.48	0.47	0.53
	0.44	0.45	0.42	0.42	0.47	0.43	0.43	0.4	0.37	0.4
Ethanol productivity (g/liter · h^−1^)	2.05	2.11	2.03	2.07	1.99	1.96	1.91	2.05	1.89	2.01
	1.54	1.52	1.47	1.52	1.62	1.53	1.44	1.48	1.35	1.49
Ethanol productivity (g/liter · g^−1^ of biomass/h)	1.13	1.15	1.06	1.03	1.16	1.41	1.47	1.5	1.41	1.35
	0.58	0.58	0.55	0.53	0.64	0.68	0.74	0.72	0.65	0.66
Initial dry cell weigh (g/liter)	0.14 ± 0.01	0.14 ± 0.00	0.15 ± 0.00	0.14 ± 0.00	0.14 ± 0.00	0.12 ± 0.00	0.12 ± 0.01	0.12 ± 0.00	0.12 ± 0.00	0.12 ± 0.00
Final dry cell weigh (g/liter)	2.72 ± 0.18	2.87 ± 0.18^*a*^	3.00 ± 0.05^*a*^	2.91 ± 0.17^*a*^	2.57 ± 0.18	2.09 ± 0.08	1.95 ± 0.16	2.05 ± 0.03	2.02 ± 0.16	2.23 ± 0.07
	2.90 ± 0.26	3.02 ± 0.02	3.02 ± 0.08	2.90 ± 0.18	2.84 ± 0.06	2.24 ± 0.03	1.96 ± 0.04	2.07 ± 0.04	2.09 ± 0.13	2.26 ± 0.11
Change in cell biomass (%)		+5.51	+10.29	+6.99	−5.51		−6.70	−1.91	−3.35	+6.70
		+4.14	+4.14	0%	−2.07		−12.50	−7.59	−6.70	+0.89
Biomass yield (g per g of glucose)	0.016	0.017	0.017	0.017	0.016	0.017	0.016	0.013	0.014	0.016
	0.016	0.016	0.016	0.016	0.015	0.013	0.011	0.012	0.012	0.013
Specific growth rate (g/liter · h^−1^)	0.076	0.076	0.08	0.083	0.071	0.058	0.054	0.057	0.056	0.062
	0.055	0.055	0.056	0.059	0.058	0.047	0.041	0.043	0.044	0.047

a *P* < 0.05 compared to FY73 (wild type).

b Parameter values are for strains grown in yeast extract-peptone-dextrose (YPD) containing 20% glucose (wt/vol), taken at start where noted or at 36 h (top line) or 48 h (bottom line) of fermentation. Results were obtained from at least two independent experiments performed in triplicate.

Regarding the fermentation profiles, glucose consumption was completed by 36 h for all strains tested (Fig. 6B). The *ZNF1-*overexpressing and *ZNF1–HSP104*-co-overexpressing strains showed increased ethanol production of 75.78 and 74.34 g/liter, respectively, representing 2.8% and 0.85% increases compared to that of the wild-type strain (73.71 g/liter). The *HSP104-*overexpressing strain showed similar ethanol production (73.09 g/liter) to that of the wild-type strain (Fig. 6B). Results suggested a positive effect of Hsp104 on ethanol tolerance and a negative effect on ethanol production under the tested conditions. Noticeably, at 48 h of fermentation, a dramatic decrease in ethanol was observed, to only 7.3 to 7.0% (vol/vol) (Fig. 6B). The *HSP104*- and *ZNF1-*overexpressing strains could rapidly utilize ethanol compared to the wild-type strain, while the co-overexpressing strain showed a lower rate of ethanol consumption, suggesting the involvement of Hsp104 and Znf1 in ethanol utilization. Thus, Znf1 enhanced ethanol production and increased ethanol tolerance when there was sufficient glucose. However, at low ethanol and glucose levels, Znf1 does the opposite and increases ethanol utilization, as previously shown (16).

Last, in addition to spot and growth assays, CFU counts were performed using the overexpression strains and some deletion mutants. *ZNF1-*overexpressing strains OE-*ZNF1^TEF^*^/Cas9^, OE-*ZNF1^TPI^*, and OE-*ZNF1^GAL^* were constructed using genetic engineering techniques, and their growth was monitored in the presence of 5 to 12% (vol/vol) ethanol using spot tests. The *znf1*Δ strain was very sensitive to increased ethanol concentrations and displayed a low growth rate compared to that of the wild-type strain in the ethanol-free medium (Fig. 6C to E). The *ZNF1-*overexpressing strains, especially strain OE-*ZNF1^TPI^*, which contained a high-copy-number plasmid for driving a strong *ZNF1* expression under the *TPI1* promoter, displayed better growth and a higher survival rate compared to those of the wild-type strain (Fig. 6C to E). qRT-PCR analysis showed variations in the *ZNF1* expression levels of OE-*ZNF1^TEF^*^/Cas9^, OE-*ZNF1^GAL^*, and OE-*ZNF1^TPI^*, with 4-, 6-, and 13-fold increases in expression, respectively, compared to that of the wild-type strain. This may explain the differential growth observed among the *ZNF1*-overexpressing strains (Fig. 6C to E). Induction by galactose also affected the growth of these strains with increasing ethanol concentrations (Fig. 6C), suggesting that Znf1 plays a key dynamic role in regulating ethanol tolerance and carbon utilization. Nevertheless, overexpression of *ZNF1* enhanced the survival rate during ethanol stress (Fig. 6C to E). Moreover, the *gpp1*Δ strain lacking glycerol-3-phosphate phosphatase and the *gpd1*Δ strain lacking glycerol-3-phosphate dehydrogenase were studied; both are key enzymes of glycerol production. Both deletion mutant strains showed low colony counts, indicating a low survival rate during ethanol stress (Fig. 6D and E). In agreement with this, deletion of *GPP* impairs intracellular glycerol accumulation, leading to poor stress tolerance (45, 46). In addition, the *lhs1*Δ strain exhibited increased sensitivity and a low survival rate under glucose and ethanol stress conditions (Fig. 6D and E) although these were less obvious in the spot test (Fig. 5E). *LHS1* is another key target gene of Znf1 that plays a role in the UPR pathway (Fig. 5B and C). Disruption of *LHS1* causes a significant delay in the translocation of carboxypeptidase Y (CPY) and defective refolding of proteins, suppressing cell viability (47, 48). Interestingly, the *smi1*Δ strain with defective cell wall biosynthesis also showed significantly impaired growth and a low survival rate, especially during ethanol stress (Fig. 6D and E) and, as previously found, in the spot test (Fig. 3E). Smi1 and Bck2 are members of the Pkc1 signaling pathway. They coactivate cell cycle progression with Swi proteins (37, 49). Deletion of *SWI4* or *SWI6* increases ethanol yield, indicating the involvement of the cell cycle in regulating ethanol fermentation in yeast (50).

### Implication of ethanol stress-responsive transcription factors of S. cerevisiae.

**FIG 7:**
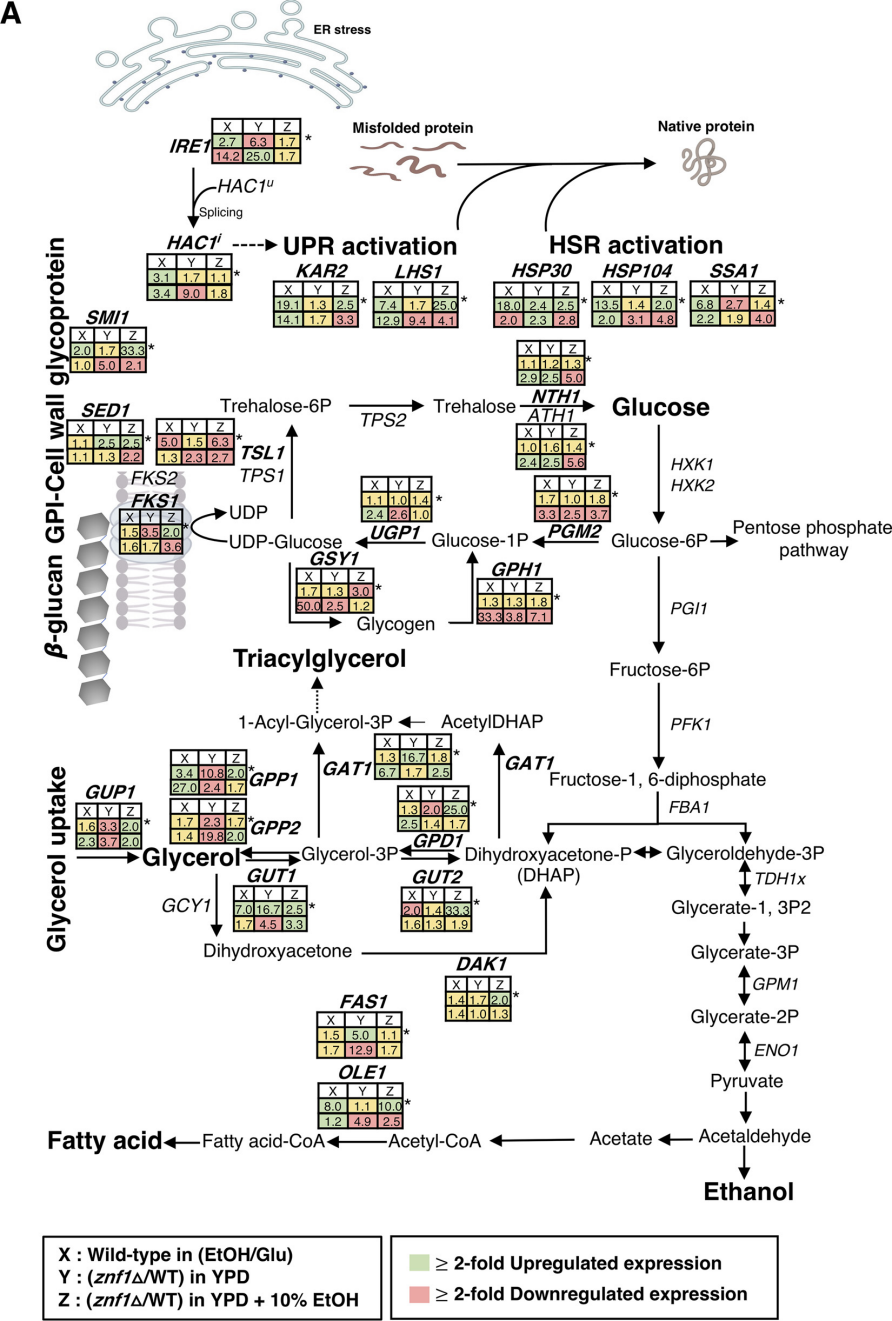
The role of the transcription factor Znf1 in ESR and its interplay with transcription factors of stress response. (A) Model of Znf1-mediated regulation of gene expression in the UPR, glycerol, carbohydrate biosynthesis, and cell wall biosynthesis in response to ethanol stress. Well-characterized gene targets of Znf1 in various pathways are displayed on the metabolic map. Changes in the levels of mRNA during high-ethanol induction are indicated in boxes labeled “X” (10% [vol/vol] ethanol induction) for the wild-type (FY73) strain, those in the *znf1*Δ strain relative to the wild-type strain in boxes labeled “Y” (2% glucose), and those in the *znf1*Δ strain relative to the wild-type strain in boxes labeled “Z” (10% [vol/vol] ethanol induction). The green, red, and yellow boxes indicate genes whose expression was activated, repressed, and unaltered, respectively, by the transcription factor Znf1 at 25 min. An asterisk (*) indicates the time point of 6 h of ethanol treatment. (B) Overlapping transcription factors and ethanol stress response genes. Venn diagram showed shared protein binding motifs with the Msn4/Msn2, Hsf1, Yap1, and Znf1 transcription factors in the promoter regions of candidate ethanol stress response genes, based on JASPAR and data from Ma and Liu (8). (C) Diagram indicating the identified putative binding sites on promoters of shared target genes of the transcription factors Znf1 (blue box), Msn2/4 (yellow box), Hsf1 (green box), and Yap1 (purple box). (D) Proposed model describing the potential mechanism and interplay of Znf1-mediated activation of key target genes involved in yeast adaptation to ethanol stress in S. cerevisiae.

Many transcription factors act in concert to promote cell tolerance to high ethanol levels (8); among them is the newly implicated transcription factor Znf1, which moderates ethanol stress response in S. cerevisiae through various mechanisms, including the activation of glycerol protectant, cell wall biosynthesis, and UPR (Fig. 7A). Despite the functional redundancy among the stress-responsive transcription factors Msn2, Msn4, Hsf1, and Yap1 in the regulation of the ethanol stress response, they appear to have overlapping and unique gene targets (Fig. 7B). Some target genes have been characterized in detail, and their binding motifs have been identified previously (8). Two dual stress-responsive transcriptional activators (Msn4/Msn2), a heat shock transcription factor (Hsf1p), an oxidative stress tolerance transcription factor (Yap1), and a transcription factor for carbon utilization (Znf1) share target genes involved in ethanol tolerance of S. cerevisiae.

Including those described in this study, a total of 75 target genes of ethanol stress-responsive transcription factors have been documented. Among them, *GUP1*, *DAK1*, *GAT1*, *GUT1*, *GUT2*, *SED1*, *GAT1*, and *SMI1* are solely controlled by Znf1 (Fig. 7B), indicating a key role of Znf1 in the regulation of glycerol and cell wall metabolism. Znf1 primarily shares targets with Hsf1 but has unique target genes involved in the uptake and utilization of glycerol and triacylglycerol formation (Fig. 7B). Znf1 shares 9 target genes with Hsf1, 6 genes with Msn2/4, and 4 genes with Yap1 (Fig. 7B). As shown, three key genes involved in protein folding, namely, ATPase gene *SSA1* of the *HSP70* family, *HSP104*, and *HSP30* are regulated by all transcription factors (Fig. 7B). Using a predictive software program, it was found that transcription factor Znf1 could directly bind to the *HSP30* promoter (Fig. 7C), indicating a vital role of these genes during ethanol stress. *HSP30* encodes the plasma membrane heat shock protein involved in responses to heat stress, osmotic stress, DNA damage, and negative regulation of ATPase activity (51). *HSP104* encodes disaggregase, which cooperates with Hsp40 and Hsp70 to refold and reactivate previously denatured, aggregated proteins in response to various types of stress (52, 53). Therefore, it is suggested that protein denaturation occurs as a result of ethanol toxicity. Together, the transcription factors coordinate and fine-tune the level of gene expression in response to ethanol stress (Fig. 7D).

## DISCUSSION

In this study, exposure to high ethanol levels led to cell wall and protein folding stress. Ethanol toxicity reduces the growth of S. cerevisiae, especially the yeast lacks the Znf1 transcription factor. Expression of Znf1 target genes linked to ethanol tolerance is actively and robustly reprogrammed and includes the activation and repression of some genes involved in carbohydrate metabolism, cell wall biosynthesis and integrity, glycerol and fatty acid biosynthesis, and the UPR pathway (Fig. 7A). First, ethanol strongly induces the expression of the *GPD1*, *GPP1*, and *GUP1* genes (glycerol biosynthesis), and their mRNA levels are strongly affected by *ZNF1* deletion (Fig. 1C). These key glycerol metabolic enzymes are required for cell membrane integrity, and high *GUP1* expression induces membrane proliferation (24). High accumulation of intracellular ethanol inhibits growth and disrupts the functions of proteins and enzymes, leading to membrane damage through alterations to cell membrane and cell wall compositions (24, 29). High ethanol concentrations could also reduce sugar assimilation and inhibit the activity of glycolytic enzymes, causing slower sugar utilization and impairing fermentation ability (54). Since the production of the main precursors of cell membrane glycerol and fatty acids largely depends on Znf1 induction (Fig. 1D and G), proper control of biosynthesis and the uptake of these metabolites is crucial to preventing ethanol toxicity. Thus, Znf1 is vital for glycerol protection of yeast cells by preventing an increase in cell membrane permeability elicited by ethanol-induced stress (Fig. 1 and 2).

However, after glucose depletion, yeast may utilize the fermentable product ethanol as a carbon source via nonfermentative metabolism (55). Znf1 chiefly controls the utilization of ethanol via activation of gluconeogenesis, thereby decreasing ethanol accumulation (16). During ethanol treatment and under high glucose concentrations, glycerol production occurs in strains lacking Znf1 and its target genes, *LHS1* or *SMI1* (Fig. 4), to compensate for *ZNF1* deletion, leading to a defective cell wall and defective cell membrane integrity (Fig. 2) (16). The deletion of *LHS1*, involved in unfolded protein response, also induces glycerol production (56). Smi1 interacts with Slt2 in the mitogen-activated protein (MAP) pathway. *SMI1* deletion causes an imbalance of Slt2-MAP, leading to reduced cell wall integrity and cell propagation, as shown by a defective phenotype following CFW treatment (57). In the event of cell wall damage, both Slt2 and the transcription factor Hog of the MAP kinase pathway are required for the maintenance of cell wall integrity. Hog is also involved in glycerol induction during stress adaptation (58, 59). Deletion of the cell wall sensor *SMI1* or the regulatory gene *ZNF1* leads to increased sensitivity to CFW (Fig. 3F) and may affect the Hog-MAP kinase pathway, which probably explains the observed glycerol production (Fig. 1D and 1I).

Second, the UPR pathway appears to be activated early during ethanol stress response to refold and repair aggregated proteins. It is well known that ethanol stress has a negative effect on cells through the accumulation of protein aggregates in the ER (60). The UPR pathway is strongly regulated by the transcription factors Hac1 and, as shown here, Znf1 (Fig. 5 and 7D). The expression of *KAR2*, *HSP104*, *LHS1*, *SSA1*, *HAC1*, and *IRE1* is induced simultaneously, suggesting a dynamic change in reprogramming of UPR gene expression under the control of transcription factor Znf1 (Fig. 5). *IRE1,* as a signal of ER stress, triggers the Hac1 transcription factor to activate UPR genes such as *KAR2* and *LHS1* (15, 61). However, Znf1 does not appear to regulate *HAC1* and *IRE1* expression, suggesting cooperative regulation between Znf1 and Hac1 rather than sequential regulation of both UPR genes.

Znf1 regulation of *HSP30*, *HSP104*, *KAR2*, and *LHS1* strongly suggests a connection between ethanol stress and ER stress, mediated by the transcription factor Znf1 (51–53, 62). *KAR2* encodes ATPase, involved in protein import into the ER, and *LHS1* is involved in polypeptide translocation and folding. This has been confirmed by the high expression of Hsp104-GFP, which indicates protein aggregation in the *znf1*Δ strain, similar to what has been reported for the *ire1*Δ strain lacking the UPR sensor (Fig. 5D). According to Li et al. (63), *HSP104* (a member of the protein quality control complex), together with the ubiquitin-proteasome proteolytic pathway, is required for protein disaggregation and degradation of misfolded proteins under ethanol stress, among others (63). Additionally, protein disaggregase Hsp104 is mediated, propagated, and transmitted efficiently to newly formed buds (64). Together with the Znf1 transcription factor, which regulates ethanol stress response genes, Hsp104 can increase yeast propagation. Meanwhile, the ethanol production profile of the *HSP104*-overexpressing strain was lower than that of the *ZNF1*-overexpression strain. This suggests that *HSP104* promotes cell propagation, while *ZNF1* supports cell survival and increased fermentation (Fig. 6 and Table 1).

In summary, as shown in Fig. 7A, Znf1 downregulated the biosynthesis of glycogen and trehalose via the *GSY1* and *TSL1* genes, respectively. This may lead to the accumulation of UDP-glucose for utilization of β-1,3-d-glucan as a key component in cell wall construction. In addition, trehalose metabolic genes and *HSP* genes also work together to respond to ethanol stress (65). In this close interplay, trehalose prevents protein denaturation as a first step, and HSPs subsequently stop protein aggregation and promote refolding into functional conformations (43, 53). The simultaneous expression of genes involved in glycogen biosynthesis, such as *GSY1* and *GSY2*, has also been observed under ethanol stress (5). Moreover, Znf1 reconfigures the cell wall architecture via three key genes, *FKS1*, *SED1*, and *SMI1*. These are important for the synthesis of 1,3-β-d-glucan and GPI-cell wall glycoproteins, involved in the regulation of cell wall synthesis and cell integrity. The high expression of *FKS1* increases β-1,3-glucan and allows cells to tolerate ethanol stress (66). Furthermore, the deletion of ethanol stress response genes involved in cell wall biosynthesis and UPR pathways also signals glycerol induction. As observed, Znf1 activates genes through transporter Gup1 that are required for the accumulation of intracellular glycerol, as well as for glycerol uptake, to remodel cellular membranes through GPI anchor proteins. In addition, high expression of *GUP1* enhances protein biosynthesis of the ER system, Golgi metabolism, and phospholipid biosynthesis for membrane reconstruction (24).

Finally, along with the roles of other well-known transcription factors, this study demonstrates a role for the Znf1 transcription factor in ethanol stress response through cross-regulation of pathways for the glycerol biosynthesis, cell wall integrity, and the UPR (Fig. 7D). Reprogramming of Znf1 target genes leads to increased intracellular glycerol and biosynthesis of cell wall components. Additionally, it prevents protein aggregation through the activation of the UPR to enhance the robustness of strains during alcoholic fermentation. Nowadays, the production of ethanol, a key biotechnological product, is crucial for food and nonfood industrial sectors, as well as for health care and pharmaceutical applications. Moreover, bioethanol is becoming popular in the automotive industry as many countries seek to attain energy efficiency, sustainability, and independence while reducing their greenhouse gas emissions. Therefore, this characterization of transcription factors implicated in ethanol stress adaptation provides important strategies for the construction of ethanologenic yeast strains that coexpress genes for alcoholic fermentation.

## MATERIALS AND METHODS

### Strains and culture media.

Yeast strains (Table 2) used in this study for phenotypic analysis were the wild-type S. cerevisiae strains FY73 (*MATα his-Δ200*; *ura3-52*) (67), BY4742 (*MATα his3Δ1*; *leu2Δ0*; *lys2Δ0*; *ura3Δ0*) and deletion mutants (68), the isogenic *znf1*Δ strain (*MATα his-Δ200*; *ura3-52*; *yfl052w*::*HIS3*) (69), and the *ZNF1*-overexpressing strain (pTEF*-ZNF1*^Cas9^, pTPI-*ZNF1* [19], and pYES2-*ZNF1* [19]). The wild-type FY73 strain and the *znf1*Δ strain were used for gene expression analysis. All S. cerevisiae strains were routinely grown in a yeast extract-peptone-dextrose (YPD) medium containing 1% yeast extract, 2% Bacto peptone, and 2% dextrose, or in YPDG supplemented with 2% galactose to induce overexpression of the *GAL* gene.

**TABLE 2:** Yeast strains used in this study

Strain	Relevant genotype and/or description	Source or reference
FY73	*MATα his-Δ200*; *ura3-52*	67
*znf1*Δ	FY73 isogenic; *znf1*::*HIS3*	69
OE-*ZNF1^TEF^*^/Cas9^	FY73 isogenic; IS7::*TEF-ZNF1*	This study
OE-*HSP104^TEF^*	FY73 isogenic; p*TEF*-*HSP104*-GFP tagged	This study
OE*-ZNF1^TEF^*^/Cas9^-*HSP104^TEF^*	FY73 isogenic; IS7::*TEF-ZNF1,* p*TEF*-*HSP104*-GFP tagged	This study
OE-*ZNF1^GAL^*	FY73 isogenic; p*GAL-ZNF1*	19
OE-*ZNF1^TPI^*	FY73 isogenic; p*TPI-ZNF1*	19
BY4742	*MATα his3Δ1*; *leu2Δ0*; *lys2Δ0*; *ura3Δ0*	Open Biosystems
*znf1*Δ	BY4742 isogenic; *znf1*::kanMX4	Open Biosystems
*ath1*Δ	BY4742 isogenic; *ath1*::kanMX4	Open Biosystems
*bck1*Δ	BY4742 isogenic; *bck1*::kanMX4	Open Biosystems
*dak1*Δ	BY4742 isogenic; *dak1*::kanMX4	Open Biosystems
*fks1*Δ	BY4742 isogenic; *fks1*::kanMX4	Open Biosystems
*gat1*Δ	BY4742 isogenic; *gat1*::kanMX4	Open Biosystems
*gpd1*Δ	BY4742 isogenic; *gpd1*::kanMX4	Open Biosystems
*gph1*Δ	BY4742 isogenic; *gph1*::kanMX4	Open Biosystems
*gpp1*Δ	BY4742 isogenic; *gpp1*::kanMX4	Open Biosystems
*gpp2*Δ	BY4742 isogenic; *gpp2*::kanMX4	Open Biosystems
*gsy1*Δ	BY4742 isogenic; *gsy1*::kanMX4	Open Biosystems
*gup1*Δ	BY4742 isogenic; *gup1*::kanMX4	Open Biosystems
*gut1*Δ	BY4742 isogenic; *gut1*::kanMX4	Open Biosystems
*gut2*Δ	BY4742 isogenic; *gut2*::kanMX4	Open Biosystems
*hac1*Δ	BY4742 isogenic; *hac1*::kanMX4	Open Biosystems
*hsp104*Δ	BY4742 isogenic; *hsp*104::kanMX4	Open Biosystems
*hsp30*Δ	BY4742 isogenic; *hsp30*::kanMX4	Open Biosystems
*ire1*Δ	BY4742 isogenic; *ire1*::kanMX4	Open Biosystems
*kre1*Δ	BY4742 isogenic; *kre1*::kanMX4	Open Biosystems
*lhs1*Δ	BY4742 isogenic; *lhs1*::kanMX4	Open Biosystems
*nth1*Δ	BY4742 isogenic; *nth1*::kanMX4	Open Biosystems
*pgm2*Δ	BY4742 isogenic; *pgm2*::kanMX4	Open Biosystems
*sed1*Δ	BY4742 isogenic; *sed1*::kanMX4	Open Biosystems
*smi1*Δ	BY4742 isogenic; *smi1*::kanMX4	Open Biosystems
*ssa1*Δ	BY4742 isogenic; *ssa1*::kanMX4	Open Biosystems
*swi6*Δ	BY4742 isogenic; *swi6*::kanMX4	Open Biosystems
*tip1*Δ	BY4742 isogenic; *tip1*::kanMX4	Open Biosystems

### Determination of multiple-stress sensitivity in yeast strains.

The sensitivity of the yeast strains, including that of the *ZNF1-*overexpressing, deletion, FY73 (wild-type), and BY4742 (wild-type) strains, was evaluated at different stressor concentrations by a spot assay. Yeast cells were cultured in YPD or YPDG at 30°C for 18 h; then, the cells were harvested and resuspended in distilled water to the same optical density at 600 nm (OD_600_) of 0.1. The cell suspension was serially diluted 10-fold (10^−4^ to 10^−1^) and kept at room temperature. Next, a 3-μl portion of each dilution was spotted onto YPD or YPDG plates supplemented with different concentrations of ethanol (5, 10, 12, and 15% vol/vol), β-mercaptoethanol (5, 10, 15, and 25 mM), and calcofluor white (0.01, 0.1, and 0.2 mg/ml). A YPDG medium containing galactose was used to induce the expression of gene *ZNF1*, constructed under the control of the *GAL* promoter. The growth of yeast cells was assessed after incubation at 30°C and 37°C for 2 to 3 days.

### Construction of a yeast strain containing *HSP104*-GFP.

Escherichia coli strain DH5α, containing the *HSP104*-GFP inserted in the URA3/CEN low-copy-number shuttle vector (70), was grown in LB medium supplemented with 0.1 mg/ml ampicillin at 37°C overnight. Plasmid DNA was extracted from E. coli cells using the Presto mini plasmid kit (Geneaid Biotech). The plasmid was digested with the restriction enzyme BamHII to confirm the correct integration of *HSP104* in the plasmid. The yeast transformation was performed using the dimethyl sulfoxide (DMSO) lithium acetate (LiOAc) method (71–73). A total of 200 μl of cells was spread on selective yeast nitrogen base (YNB)-Ura medium containing 0.67% yeast nitrogen base, 2% glucose, 0.082% yeast synthetic drop-out medium, minus Ura (Sigma), and 2% Bacto agar.

### Fluorescence microscopy.

The Fluoview FV10I confocal laser scanning microscope (OLYMPUS, Germany) was used to obtain live-cell images of yeast. Ethanol stress was induced by cultivating cells in YPD medium containing 10% (vol/vol) ethanol at 30°C or at 37°C for 25 min. Then, cells were fixed with 3.5% formaldehyde and washed before examination under the microscope.

### Gene induction and quantitative real-time PCR.

The FY73 (wild-type) and *znf1*Δ strains were grown in YPD at 30°C overnight until an OD_600_ of 0.1 and regrown to an OD_600_ of approximately 0.6. Then, 10% (vol/vol) ethanol was added to cell culture, and sampling was performed at 25 min and 6 h. For growth under high-glucose conditions, the BY4742 (wild-type) and *znf1*Δ strains were grown in YPD at 30°C overnight. Next, cells were regrown to the mid-log phase and transferred to YP medium containing 20% glucose (wt/vol) for additional 24 h. Yeast cells were harvested and washed twice using distilled water. Then, the total RNA was extracted by the phenol-chloroform method (74) and purified using the RNeasy minikit (Qiagen, Hilden, Germany). cDNA synthesis was carried out with the qPCRBIO cDNA synthesis kit (PCR Biosystems, USA). qRT-PCR assays were performed using a CFX Connect real-time PCR detection system with the CFX Manager software for analysis. The reaction mixtures contained qPCR master mix (New England Biolabs [NEB], USA). Samples without reverse transcriptase or nontemplate controls were included in the qRT-PCR analysis. The relative quantification of each transcript was calculated using the threshold cycle (2^−ΔΔ^*^CT^*) method and normalized using the *ACT1* gene as an internal control (75). Sequences of primers used for qRT-PCR are listed in Table 3.

**TABLE 3:** Primers for RT-qPCR analysis and strain construction used in this study

Purpose or pathway	Gene/primer	Primer sequence(s) (5′ to 3′)
RT-qPCR	*ACT1*	ATTATATGTTTAGAGGTTGCTGCTTTGG and AATTCGTTGTAGAAGGTATGATGCC
	*ZNF1*	AGGCACTAATTGATCAGTGTCTGC and GCAGAAACTGGATAACTGTATCC
UPR pathway	*HAC1*	ACTGAACAGCGTCAACCTTG and GTAGCGTCGTCGACTCTG
	*HSP30*	GCAAGTCTATCACAGGTG and AACATAGCGACAGCACCA
	*HSP104*	TGCACCTGCGGAGATAAC and GCGGTCTTACCGATACCTG
	*IRE1*	GATATACTAATCGCAGCCGACG and GTAACCCTTGATGGGCGT
	*KAR2*	CGTGGCATTCACCGATGA and GATGGTACCAGCATCCTTGG
	*LHS1*	CTATATTGCAGCCGGAGGAC and GCCTCAGAATTGGCGCTTA
	*SSA1*	AGGCTGACATGAAGCACTTC and AGCATCCTTGGTAGCTTGTC
Glycerol metabolism	*DAK1*	TATTAACTGCCGCGTTGC and TTAGCTGTACCGTCTAAGCC
	*GAT1*	TGCATAACCGCACGTCTT and GAGCCAGGCTTCACTATAGATG
	*GPD1*	GTGGGTGTTCGAAGAAGAG and ACAGGAGATAGCTCTGACG
	*GPP1*	GAAGTTCCAGGTGCTGTC and CGTTGTATTCACCGACTC
	*GPP2*	CCGGTCAAGTACGGTGAA and CAATACCTGCTGGAGCGT
	*GUP1*	GAACGTCTCCTATCTCAAGG and TGCTGATGGTGGCTATTC
	*GUT1*	CTGCAGAAGGCTATGCCA and CCTTGTAAGGTGGGAGACC
	*GUT2*	TCGACGTGTTGATCATCGG and AATTGGTAGCACCGTGCA
Fatty acid biosynthesis	*FAS1*	CTAGTCGAACCTTCCAAGGTC and GTGTTACCTTGACCACCGA
	*OLE1*	CATCTCCGAACAACCATGGA and ATCTCAATGGCCAGTGAGC
Carbohydrate metabolism	*ATH1*	ACATACTCACATGCCTCGAG and CTAAGGCATAGCCGAACC
	*GPH1*	GCTTATGAAGCTGCTTCG and CGGTTCTTGGTCCAAGACA
	*GSY1*	CGAATGGAAGGCTGACCTA and ATGCCTCTCTTACCAGCTTC
	*NTH1*	ACAGACTAGACGTGGTTCTG and CGGTATCCTCGATGGTCA
	*PGM2*	AGATTGCCGCTATCGGTG and TTGTGACGGATTCAGGAGC
	*TSL1*	GCGAATGCAACTACCTCACA and GTTGCTGTTGGATTGGCG
	*UGP1*	CAGTACGATAGCGACGTGC and GTGACCTGGTGGATACCAAG
Cell wall biosynthesis	*FKS1*	ATATGGCTGCTCAAGACGG and GAGGACCTAGAGTCCAAGAGAA
	*SED1*	CGGTACTTCTACTGAAGCTCC and CAGTGACGGTGTAAGTCTTACC
	*SMI1*	ATCACTCACGCAGAGGAAGA and GTGCATATACCGGTTGCACT
Strain construction	D1306	CAAGAACTTCGTATCGGCTTTC
	D1307	GCTAGAGACATGTTAGAAGAC
	D1308	CATTAGCAGAGTCGGTAGCTA
	D1309	TTCTAGAACTAGTGGATCCATGGCCCGCAATAGACAAGCG
	D1310	AACTAATTACATGACTCGAGTTAAGGAAGCGCATCTACATC
	D1321	GATCATCATAACACAGCAAAGGC
	C3586 and C3587	CAAAGGTTGATGCAAGTCGA and GTAATCCCTTCCACCTTTCT
	C4330 and C4331	GGAGCAGACATCACTAAACG and GCCACAACCAAGTGAGATAC

### Construction of *ZNF1* overexpression strain via the CRISPR/Cas9 system.

DNA was extracted from the yeast strain FY73, followed by the amplification of *ZNF1.* The PCR was performed with Q5 high-fidelity DNA polymerase (catalog no. M0491; NEB) and primers (D1309 and D1310). The PCR products were analyzed, purified, checked by gel electrophoresis and NanoDrop, and sent for sequencing. Primers used for sequencing (D1306, D1307, D1308, and D1321) are listed in Table 3. For cloning, first, the p426hph-AD7 plasmid was linearized with BamHI and Xhol restriction enzymes. The cut plasmid and the amplified *ZNF1* PCR fragment were ligated using the NEBuilder HiFi DNA assembly cloning kit (NEB) and incubated at 50°C for 1 h. After transformation, selection was done using the ampicillin marker, and the positive clones were checked for *ZNF1* insertion in the p426hph-AD7 plasmid by colony PCR assays. PCR was performed using primers (C3586 and C3587), and the PCR product was cleaned using the Gel and PCR clean-up system. Primers for sequencing were C3586, C3587, D1306, D1307, D1308, and D1321, as listed in Table 3. The cells were transformed with the *CAS9* plasmid (p51) and p426hph-AD7-*ZNF1*(p2964-*ZNF1*) using the LiOAc method (73), and then used for template TEF-*ZNF1*-CYC as a donor. The yeast-Cas9 cell was cultured in YPD medium at 30°C for 18 h. Competent cells (50 μl) were mixed with 4 μl of 500 ng guide RNA plasmid with selectable markers, nourseothricin, and 4 μg of the donor DNA; 10 μl of single-stranded DNA (ssDNA); and 300 μl of PLI (1 M LiAc and 50% polyethylene glycol [PEG] 3380), as described previously (73). Cells (100 μl) were spread on selective YPD plates containing 100 mg/liter Geneticin for Cas9 and 100 mg/liter nourseothricin (nourseothricin *N*-acetyl transferase [NAT]). The remaining cells were centrifuged, spread on the selective medium, and incubated at 30°C. Colonies were then examined via PCR for *ZNF1* insertion in chromosome AD7. DNA was extracted from cells and amplified by PCR using *Taq* DNA polymerase and primers C4330 and C4331 (Table 3). The PCR product was analyzed on a 0.8% agarose gel in 0.5× Tris-acetate-EDTA (TAE), after which sequencing was performed.

### Plasma membrane integrity assay.

Yeast cells were precultivated in YPD until an OD_600_ of 0.2 and regrown to an OD_600_ of approximately 0.6. Then, cells were diluted to an OD_600_ of 0.2. After that, cells were incubated in YPD supplemented with 10% (vol/vol) ethanol for 25 min and 6 h. Yeast cells were harvested and washed three times with phosphate-buffered saline (PBS) containing 0.01% Tween 20 (vol/vol) (PBST). Then, cells were treated with 5 μg/ml propidium iodine (PI) staining solution (Sigma-Aldrich) and incubated at 30°C for 30 min in the dark (76). To evaluate the plasma membrane integrity, cells were examined under a fluorescence microscope (BX53 upright microscope; Olympus). The maximum fluorescence excitation was 535 nm and maximum emission was 617 nm. The PI-positive percentage was calculated from two independent experiments performed in triplicate.

### Intracellular content measurement.

Cells were cultured in 50 ml of YPD until they reached an OD_600_ of approximately 0.6. Then, cells were split in half into two separate cultures. A final concentration of 10% (vol/vol) ethanol was added to one culture, and then cells were regrown and collected at 25 min and 6 h, as indicated. The intracellular glycerol of yeast cells was examined according to Petelenz-Kurdziel et al. (77). The cells were centrifuged, and the cell pellets were boiled for 10 min and collected. The concentrations of intracellular contents were measured using high-performance liquid chromatography (Dionex) with an Aminex HPX-87H ion exchange column (Bio-Rad, Hercules, CA). The analysis was performed with 5 mM H_2_SO_4_ as the mobile phase at a flow rate of 0.6 ml/min and an oven temperature of 50°C.

### Fermentation.

The wild-type, deletion mutant, *ZNF1*- or *HSP104*-overexpressing, and *ZNF1–HSP104*-co-overexpressing S. cerevisiae strains were cultured in 50 ml YPD broth in 250-ml flasks and incubated overnight at 30°C with shaking at 150 rpm. Cells were diluted to an OD_600_ of 0.1 or 1.0 and then transferred into 50 ml of YP broth with 2 or 20% (wt/vol) glucose, respectively. The flasks were incubated at 30°C with shaking at 100 rpm. Cell samples (1.5 ml) were harvested at 0, 12, 24, 48, 72, and 96 h. The glucose, ethanol, and glycerol concentrations in the medium were analyzed by HPLC (Shimadzu, Japan) with an Aminex HPX-87H ion exchange column (Bio-Rad) to determine the concentration. A mobile phase of 5 mM H_2_SO_4_ was used at a flow rate of 0.6 ml/min, and the column temperature was 65°C. Extracellular content of metabolites was measured during fermentation with 2% (wt/vol) glucose only.

### Determination of cell growth and cell survival under ethanol stress.

S. cerevisiae wild-type (FY73 and BY4742), *znf1*Δ, *ZNF1*-overexpressing, and deletion mutant strains were routinely grown in YPD. These strains were precultured at 30°C overnight with shaking. Aliquots of cells were then used to inoculate fresh YPD and regrown at 30°C with shaking to an OD_600_ of 0.6. Then, the culture was divided in half and treated with and without a 10% (vol/vol) final concentration of ethanol. Culture samples were taken at 0, 4, 6, 24, 48, 72, and 96 h to measure OD_600_ by spectrophotometry to construct growth curves. In parallel, cells collected at each time point were spread on YPD agar and incubated at 30°C for 2 days to determine cell survival by CFU count. At least two independent experiments were performed in triplicate.

### Construction of Venn diagram for ethanol response.

The protein-binding motifs of gene targets of transcription factors (Msn4/Msn2, Hsf1, Yap1, and Znf1) were predicted using JASPAR (http://jaspar.genereg.net/) (78) and YEASTSTRACT (http://www.yeastract.com/) (79). Previous data on ethanol tolerance in yeast (8) were referenced for the construction of the Venn diagrams.

### Statistical analysis.

Analyses were performed using SPSS Statistics for Mac v. 26 (IBM Corp., Armonk, NY) using Student’s *t* test, and a *P* value of <0.05 was considered statistically significant. At least two independent biological experiments were performed with at least three technical replicates.

### Data availability.

Data will be made publicly available upon request.
